# The Obstetrical Remembrancer; or Denman’s Aphorisms on Natural and Difficult Parturition; the Application and Use of Instruments, &c.

**Published:** 1848-06

**Authors:** 


*The Obstetrical Remembrancer ; or Denman's Aphorisms on Natural and difficult parturition ; the application and use of Instruments, &c.* Augmented by MICHAEL RYAN, M. D. First American from the Ninth London Edition ; with additions by THOMAS F. COCK, M. D., Visiting Physician of the New York Lying-in Asylum. 18mo. pp. 264. Samuel S. & William Wood. New York, 1848.

This is a very small book to claim so numerous a paternity. The basis of it may be said to be very old, too, for a work on science, but the excellence of the matter has lost nothing from the progress of time, and on most points it will be found quite as orthodox as the works of more recent date. "Denman's Aphorisms," ever since their first publication, have been regarded as safe guides for the young obstetrician, and useful monitions for the old.

The contributions by the American editor, although few and brief, are sound and practical.

If every young practitioner were to carry this little volume about him as a *pocket-book*, for which its size so well adapts it, and read it during his hours of attendance on tedious cases of labour, we should see and hear less of mal-practice than some of us, alas! are compelled to witness.

